# Editorial: Current and Future Role of Artificial Intelligence in Cardiac Imaging

**DOI:** 10.3389/fcvm.2020.00137

**Published:** 2020-08-07

**Authors:** Karim Lekadir, Tim Leiner, Alistair A. Young, Steffen E. Petersen

**Affiliations:** ^1^Universitat de Barcelona, Artificial Intelligence in Medicine Lab (BCN-AIM), Departament de Matemàtiques and Informàtica, Barcelona, Spain; ^2^Department of Radiology, Utrecht University Medical Centre, Utrecht, Netherlands; ^3^School of Biomedical Engineering & Imaging Sciences, King's College London, London, United Kingdom; ^4^Barts Heart Centre, Barts Health NHS Trust, London, United Kingdom; ^5^NIHR Barts Biomedical Research Centre, William Harvey Research Institute, Queen Mary University of London, London, United Kingdom

**Keywords:** artificial intelligence, cardiac imaging modalities, big data, cardiac image analysis, cardiovascular personalized medicine, AI adoption and translation

## Introduction

Cardiovascular disease is currently the most common cause of morbidity and mortality worldwide (1) and thus remains an important focus for both biomedical and technological research. In the age of personalized medicine, cardiac imaging is expected to play an important role to enable more accurate and advanced quantification of structural and functional changes due to cardiovascular disorders. However, despite advances in cardiac imaging modalities, such as echocardiography, cardiovascular magnetic resonance, cardiac computed tomography, and nuclear cardiology, the heart remains a challenging anatomical organ to image and assess compared to other organ systems. The main challenges faced by cardiac imaging include the perpetual cardiac and respiratory motions, the complex geometry of the ventricles, atria and arteries, the oblique orientation of the heart with respect to the body, and the small size of some of the cardiac structures, such as the coronary arteries, trabeculae and papillary muscles, as well as the large variability in imaging conditions and protocols (including non-contrasted and contrast-enhanced cardiac imaging sequences).

Consequently, advanced tools are needed to optimize the use of cardiac imaging and to support clinicians throughout the whole value-chain of cardiovascular practice, including improved image acquisition, automated cardiac quantification, cardiac tissue characterization, imaging biomarker discovery, and clinical decision support. In this context, artificial intelligence (AI), including machine learning and computer vision, has emerged as one of the most promising topics over the last 5 years. Combined with the exponential increase in computing power, AI provides unprecedented opportunities to leverage the available collections of cardiac imaging data for developing more robust cardiac image analysis algorithms, to uncover currently unknown clinical knowledge on cardiac health and disease, and to build novel software tools that will impact clinical cardiology. This area is expected to benefit from the current efforts to provide access to large-scale and high-quality image data for the scientific community. In the US, for example, existing studies such as the Multi-Ethnic Study of Atherosclerosis and the Framingham Heart Study have for a long time compiled thousands of cardiac images. More recently, in the United Kingdom, the UK Biobank has been acquiring cardiac MRI images from tens of thousands of individuals (2). In Europe, the euCanSHare project funded by the European Commission is developing a data sharing and analytics platform to facilitate access to large-scale cardiac imaging and non-imaging data from multiple centers (www.eucanshare.eu). Similar initiatives and large cohorts are expected to emerge across the globe in the years to come, which will further enhance the potential of AI in cardiac imaging and clinical cardiology.

To promote and guide further research and developments in AI for cardiac imaging, several experts and leading institutions in the field have recently published position and review papers that outline the initial achievements, discuss the current challenges and identify future perspectives. For example, Dey et al. summarized the most promising AI methods for cardiac imaging by distinguishing between the use of classical AI and advanced approaches (3). Al'Aref et al. reviewed some clinical applications of AI in cardiac imaging by considering each cardiac imaging modality separately (4). Litjens et al. focused on the sole application of deep learning methods for cardiac image analysis (5). Finally, Petersen et al. outlined the current challenges and emerging opportunities (6), emphasizing the importance of addressing non-technical aspects of AI in cardiac imaging such as patient acceptance, data protection and AI regulation.

While these papers provided an overall presentation and promotion of the field, the goal of this special issue entitled “current and future role of AI in cardiac imaging” is to compile more detailed and focused reviews covering the whole value-chain of AI in cardiac imaging. Specifically, we have invited active experts across the globe to submit in-depth reviews on several keys areas of AI in cardiac imaging, including (1) cardiac image reconstruction, (2) cardiac image segmentation, (3) cardiac shape and motion analysis, (4) computer-aided diagnosis, (5) imaging-genetics integration, and (6) socio-ethical impact and regulations. This comprehensive special issue, totaling nearly 1,000 references from the field, will constitute an unprecedented resource for researchers, both novice and experienced, to study in detail the methods, applications, strategies, datasets, tools, hypotheses, limitations and opportunities that are of direct relevance to each aspect of AI in cardiac imaging. Importantly, for each paper, we requested the authors to provide descriptions and discussions for both AI and clinical audiences, to enhance the democratization and promotion of AI in cardiac imaging, and thus future collaborations and developments in the field.

## Special Issue Content

**Figure 1:**
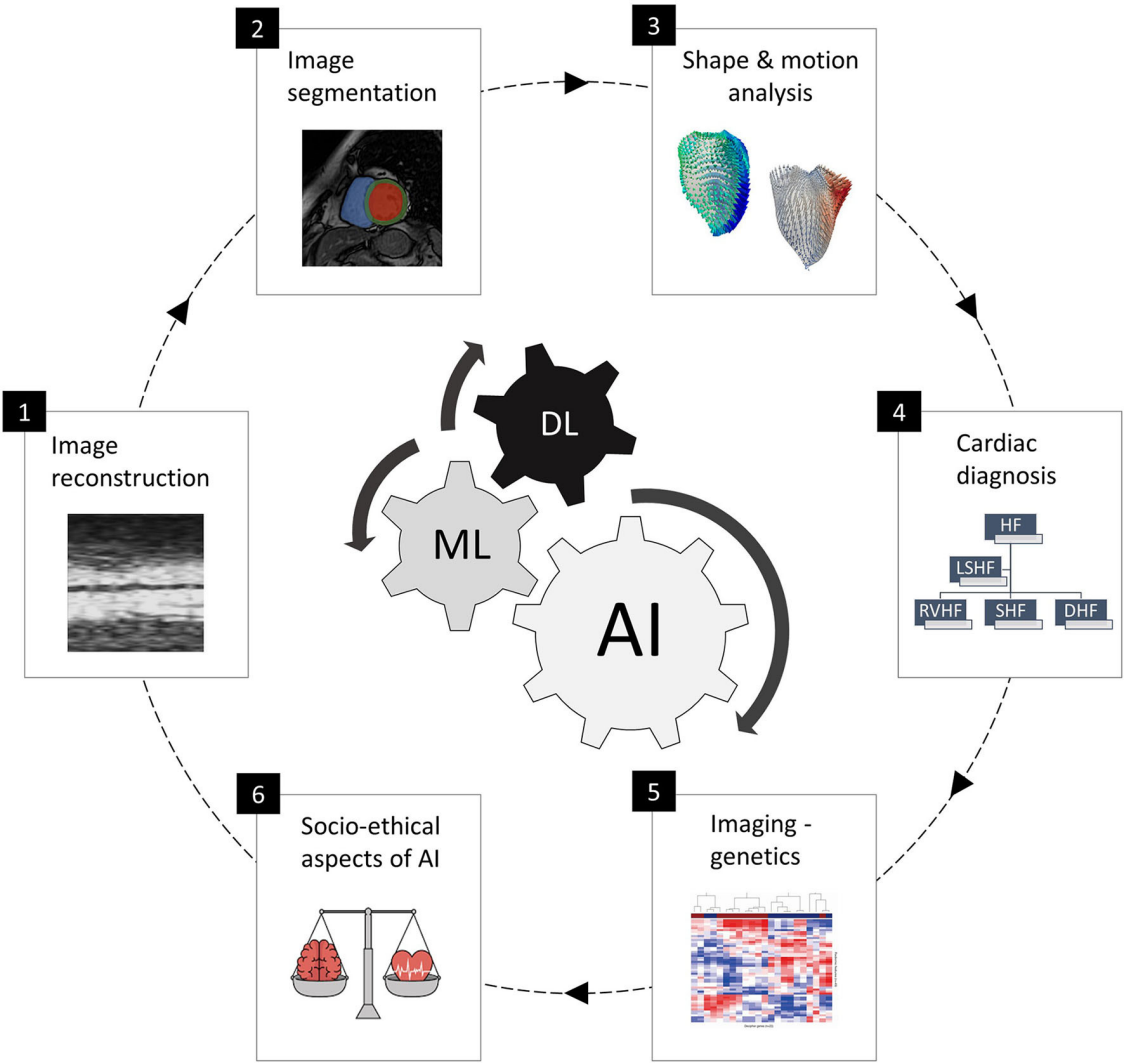
The six areas of AI in cardiac imaging covered in this special issue, representing the full clinical workflow and including cardiac image acquisition and segmentation, shape and motion estimation, image-based cardiac diagnosis and imaging-genetics studies, as well as socio-ethical and regulatory aspects of AI. The subfigures are taken from the different papers in this special issue.

This special issue cover six specific areas and application domains of AI in cardiac imaging, as shown in Figure 1 and presented as follows:

### Cardiac Image Reconstruction

The first paper of this special issue focuses on the very first step of the cardiac imaging workflow, i.e., enhancing cardiac image acquisition using AI. Fast and portable cardiac imaging such as echocardiography inherently suffers from low image quality, while high resolution cardiac imaging such as CMR requires long acquisition times to address the cardiac and respiratory motions, as well as the need to highlight the different types of cardiac structures, tissues and vessels. For a long time, enhancing and accelerating image acquisition for modalities such as CMR was targeted by developing new CMR imaging/physics sequences and techniques, such as efficient pulse sequences, motion compensation techniques, multiple radio-frequency receiver coils for parallel imaging and compressed sensing. In this special issue, Bustin et al. thoroughly surveyed emerging AI techniques for accelerating and enhancing CMR image reconstruction. Concretely, AI provides a unique opportunity to perform CMR acquisition using under-sampling strategies that acquire less image data than needed, followed by learning-based estimation of the sparse domain from existing data. The authors reviewed first the initial learning-based techniques based on dictionaries of transforms (from low to high resolution domains) learned from the acquired under-sampled data itself. Subsequently, they focused their attention on recent deep learning-based approaches, which learn the reconstruction transforms from low-resolution to high-resolution images offline based on training data. They surveyed in detail the many advances over the last 2 years, describing recent applications to both 2D dynamic cardiac imaging and 3D whole-heart CMR imaging. As deep learning based CMR reconstruction is novel as well as popular, they concluded their review by discussing the future avenues to improve real-world validation, including assessment of reconstruction quality and generalization.

### Cardiac Image Segmentation

In the next review papers, this special issue addressed the next stage of cardiac image analysis, namely cardiac image segmentation, which is by far the most covered topic by AI researchers in cardiac imaging. Deep learning approaches have been particularly popular as they have shown to generate highly accurate results when trained on large manually segmented data. At the same time, clinicians have been highly receptive to such automated black-box methods as they accelerate their clinical work without interfering in the decision making. In this special issue, Chen et al. put together a comprehensive review of deep learning techniques for cardiac image segmentation, totaling over 100 papers describing applications to various imaging modalities (echocardiography, MRI, and cardiac CT) and to the main cardiac structures (ventricle, atria, and vessels). They concluded that there is no universally optimal deep learning implementation for cardiac image segmentation, and suggested that algorithms need to be customized and optimized for each application depending on the imaging modality, protocol (e.g. contrast vs. non-contrast), and cardiac structure (left vs. right ventricle). For the immediate future, they discussed the importance of developing segmentation methods that can generalize well across various imaging modalities, scanners, and pathologies. Finally, in order to encourage reproducible research, the authors provided a summary of public datasets for training and testing new deep learning models, as well as public code repositories that include recently developed techniques.

In a more focused review, Jamart et al. surveyed one of the most challenging cardiac image segmentation applications, namely atrial segmentation from late enhanced cardiac MRI. This contrast enhanced imaging sequence is the technique of choice in clinical practice to quantify fibrosis and assess atrial fibrillation. However, the task is complicated by the geometrical complexity and small size of the atrial chambers, which are also constrained by thin walls. Moreover, the anatomical boundaries on the late enhanced MRI often lack clear contrast, which can further mislead the segmentation algorithms. As a result, until the advent of deep learning, very few techniques had been attempted for automated segmentation of the atria. In this review, the authors summarized the recent deep learning developments in this field, including multi-stage and multi-scale conventional neural networks to address the image class imbalance that is inherent to atrial segmentation (the atrial cavity represents only a small fraction of the image volume), as well as to provide contextual cues to better differentiate the atrial boundaries from the surrounding structures. The best reported performance in the survey reached a 93.2% segmentation accuracy, which is highly promising given the complexity of the task. Arguably, the most important future work in this domain is the automated detection of the fibrosis using advanced AI approaches, which will represent an important Research Topic in the years to come.

### Cardiac Shape and Motion Analysis

Image segmentation of the cardiac boundaries as described above is a pre-requisite in clinical practice to estimate standard clinical indices such as chamber volumes and ejection fraction for cardiac assessment. However, existing research has shown that more detailed information about cardiac geometry and regional motion is expected to improve future clinical assessment of normal and abnormal cardiac (dys)function. In this special issue, Gilbert et al. presented a review of the so-called statistical atlases of cardiac anatomy, which are widely used to model complex shape and function variability across subpopulations, and thus to extract new descriptors of cardiac geometry of relevance to cardiac health and disease. In this paper, the authors firstly describe the existing and newer methods for building clinically useful statistical cardiac atlases from annotated cardiac contours. Subsequently, the paper discussed that, powered by supervised or unsupervised machine learning algorithms, statistical cardiac shape analysis can be used to automatically identify and quantify abnormal shape deviations, and to provide morphometric indices that are optimally associated with clinical factors.

In another review, Duchateau et al. described in great depth the literature on cardiac motion quantification and analysis based on machine learning. With these techniques, the idea is to learn new advanced representations and patterns of myocardial motion and deformation (displacement, velocity, deformation, torsion, strain) from representative samples of cardiac images, such as echocardiography or MRI. In particular, two families of approaches were reviewed, namely (1) traditional techniques that apply machine learning onto explicit features of myocardial motion/deformation (displacement fields calculated from the images), and (2) more recent approaches based on neural networks applied directly to the image data to extract and analyze new spatiotemporal signatures from local image patches around the myocardium. In this paper, the authors described the entire workflow of steps and methods required to derive physiologically meaningful and clinically useful analyses of cardiac motion. Finally, they discussed the next steps toward clinical adoption of machine learning based cardiac motion quantification, including community benchmarking, standardization initiatives, and clinical interpretability of the extracted spatiotemporal signatures for abnormality localization.

### Computer-Aided Diagnosis

Generally, the main AI developments in cardiac imaging have mostly addressed cardiac image analysis tasks before clinical decision making, with the aim to enhance cardiac image acquisition, facilitate cardiac image segmentation, and estimate advanced indices of cardiac shape and function. However, AI is also expected to impact clinical decision making in the future, such as to enable earlier and more precise diagnosis, as well as treatment planning and response estimation. To illustrate this, this special issue includes two review papers centered on cardiac diagnosis. First, Martin-Isla et al. surveyed in detail the area of image-based cardiac diagnosis using machine learning. This included a step-by-step description of the techniques for building and validating new AI models of cardiac diagnosis. The authors also described emerging techniques for more precise diagnosis, including radiomics (omics for radiology) (7). The authors then reviewed more than 100 papers on AI- and image-driven diagnosis of coronary heart disease, cardiomyopathy, heart failure, and valve disease. Interestingly, the survey showed that some complex cardiac diseases are yet to be extensively investigated by the AI community, such as atrial fibrillation. Finally, the paper discussed current obstacles that limit the applicability of AI-driven diagnosis in cardiac imaging, in particular the lack of interpretability, which must be addressed in the years to come to enable clinicians to understand and trust the AI-generated diagnoses and decisions.

In a second review paper on AI for cardiac diagnosis, Hampe et al. focused on the assessment of coronary artery disease from non-invasive CT using machine learning. In clinical practice, catheter-guided X-ray angiography and intravascular ultrasound provide detailed information on coronary stenosis and plaque composition, but they are limited by their invasive nature. CT imaging provides a promising alternative but offers reduced contrast between the atherosclerotic plaque constituents, and thus AI is expected to play a role for the extraction of detailed and clinically useful information from the non-invasive images. This review surveyed classical and modern machine learning methods to estimate coronary stenosis, to discriminate between calcified, non-calcified and mixed plaques in CT, and to characterize fibrous and lipidic plaque constituents. Furthermore, the paper described recent methods, in particular deep learning based, for predicting fractional flow reserve directly from CT, by learning the relationship between CT features and fractional flow reserve based on a training sample of corresponding CT and invasive imaging. In their discussion, the authors noted an important limitation of current models, which are based on small training samples, as images with manually characterized plaques are more difficult to obtain than, for example, manual annotations of cardiac boundaries. Thus, this field of AI in cardiac imaging is expected to further develop for future clinical use as additional and larger datasets become available in the years to come.

### AI Integration With Non-imaging Data

To realize the promise of precision medicine in cardiology, cardiac imaging is a central piece of the puzzle. However, non-imaging data play an important role, in particular—omics and health data, as they allow to build multi-scale AI models that integrate patient-specific biomolecular, phenotypic, environmental, and clinical information. Such integrated AI models are expected to lead to improved diagnosis and treatment selection, as well as to better clinical outcomes. This special issue included an interesting review by de Marvao et al. of AI-driven integrated cardiac imaging-genetics studies, which aim to characterize the complex interplay between cardiac imaging phenotypes, environmental and genetic factors. Several concrete examples of AI-empowered imaging-genetics studies were provided, such as to enable more stratified diagnosis of heart failure, predict treatment response in cardiomyopathic patients, identify genetic variants or proteomic signatures of high-risk atherosclerotic plaques, or predict positive cardiac remodeling after cardiac resynchronization therapy. While the use of AI in cardiovascular imaging-genetics is shown to have great potential, the review noted that the challenges of AI in genetics and imaging separately are amplified by combining these very large data. Thus, further research is expected to address more ambitious whole-genome and high-resolution whole-heart imaging studies, and to derive multi-scale AI solutions for clinical practice integrating imaging, biological and clinical data.

### Ethical, Social, and Political Issues

While the review papers described above dealt with technical and clinical aspects of AI in cardiac imaging, this special issue concludes with a paper by Fenech and Buston that reviews the ethical, social, and political issues that are being investigated to facilitate future acceptance and deployment of the AI solutions in cardiac imaging. These include clarifying the impact of AI solutions on the roles of cardiologists, radiologists, and other doctors, future liability of clinicians vs. AI manufacturers, as well as on the altered relationships between healthcare professionals, patients, their relatives, and administrators. Data sharing and privacy issues were also are reviewed in the paper, focusing on the challenges to manage patient informed consent for AI solutions that remain difficult to understand (and trust) by the general public and clinicians alike. From a social point of view, initial studies reviewed in this paper suggest that there is a concern that AI solutions may remain biased and in fact exacerbate health inequalities. Furthermore, the authors discussed the need to include patients and citizens in the AI development process, to take into close consideration their requirements, expectations, and behaviors. Other important issues, such as algorithmic transparency, fairness, and regulation, were also discussed at length. For addressing these key issues and to optimize adoption by clinicians, patients, and regulators, the paper emphasized the importance of developing principles and translating them into policies in the years to come. In the general context of AI, imaging and cardiology in particular are expected to play an important role, as exemplified by the fact that they have been the healthcare domains with the greatest number of FDA approvals for novel data-driven technologies in the recent years.

## Future Perspectives

**Figure 2:**
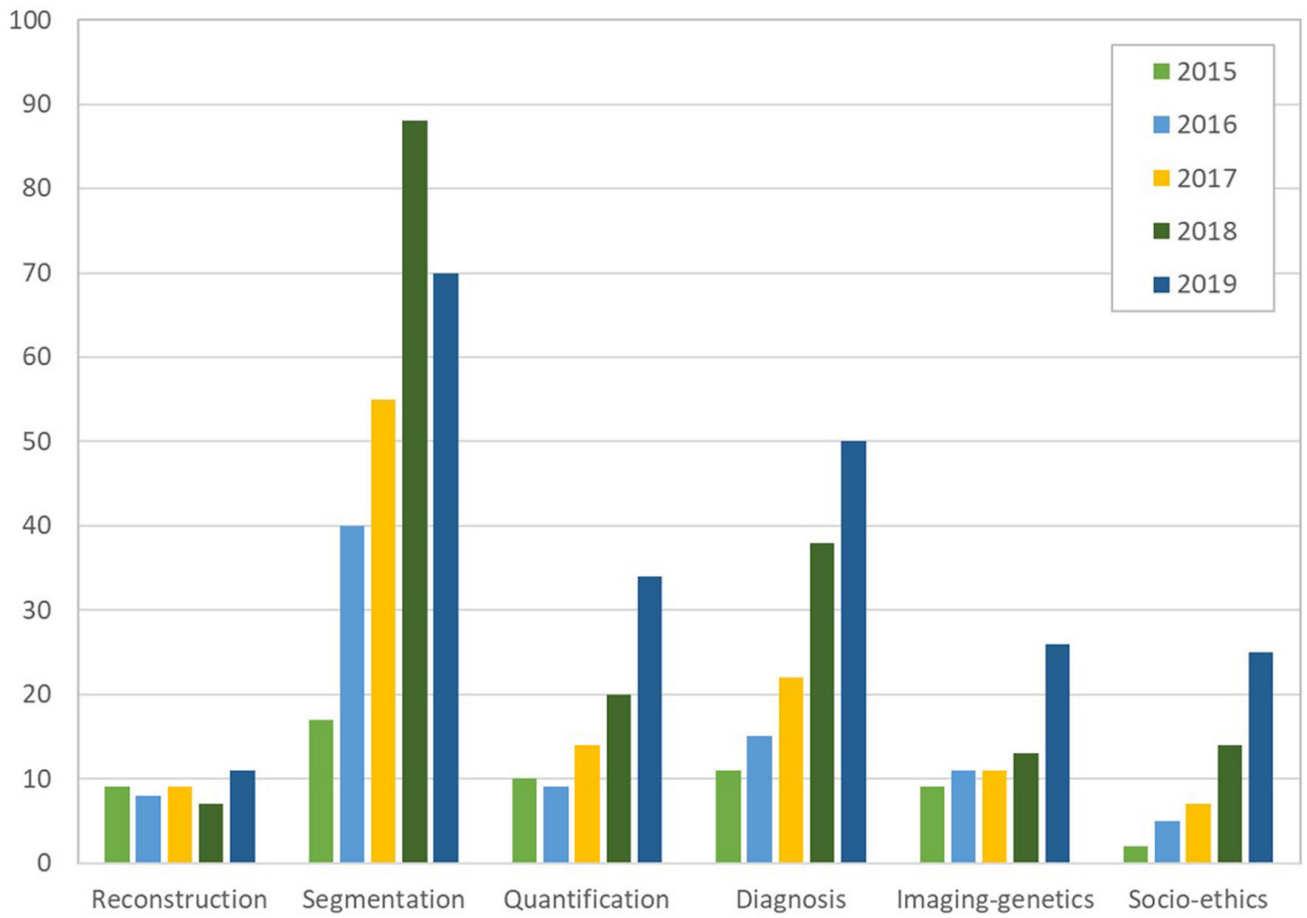
Number of papers reviewed in the six categories of cardiac imaging tasks in the last 5 years (2015 to 2019), showing an increase in the research output continuously and for all tasks, except for segmentation which decreased between 2018 and 2019 (over 550 papers reviewed in total). The subfigures are taken from the different papers in this special issue.

**Figure 3:**
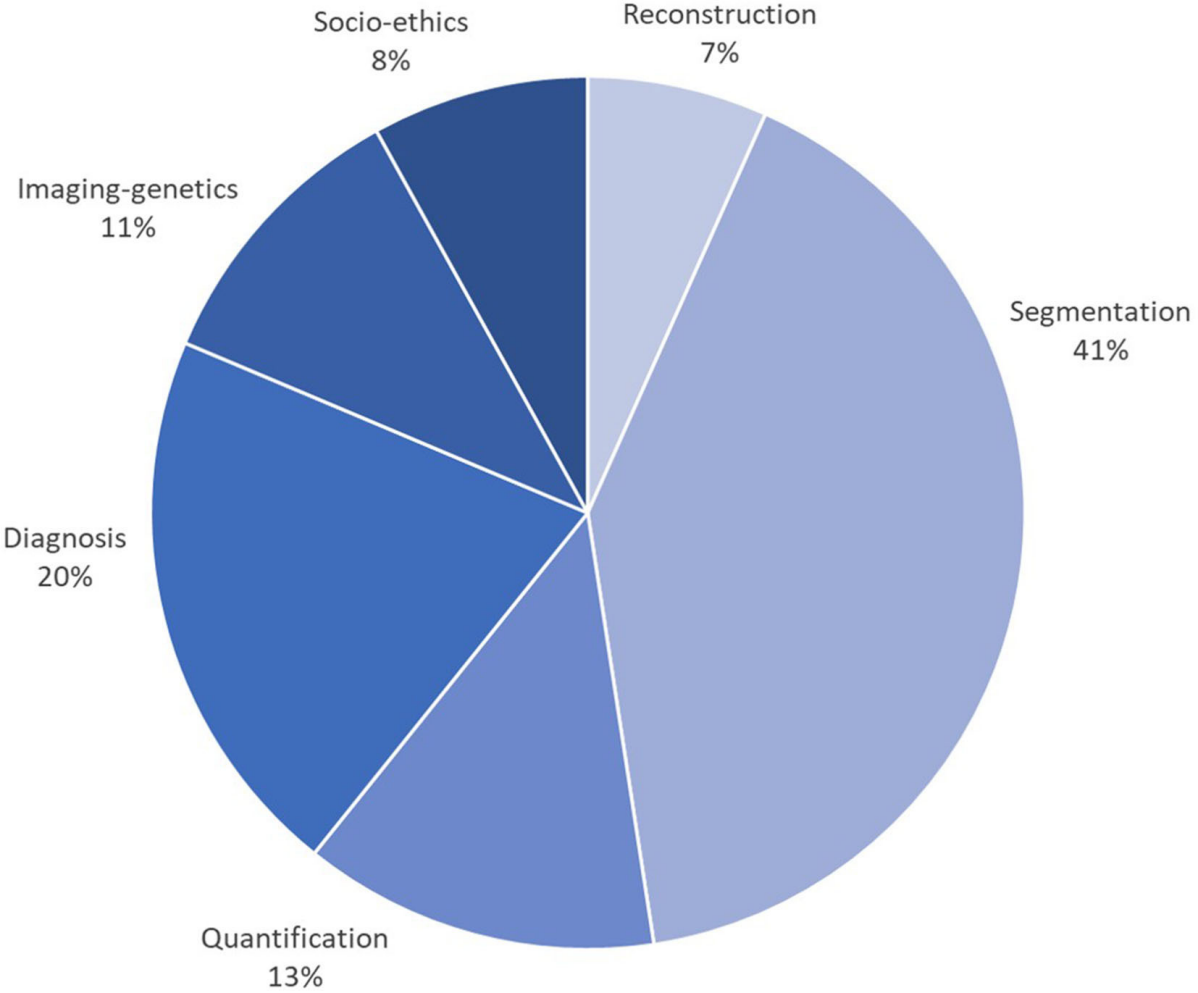
Percentage of papers surveyed per cardiac imaging task, showing that approx. 40% of efforts have been dedicated to segmentation, followed by diagnosis (20%), then shape and motion quantification (13%). The subfigures are taken from the different papers in this special issue.

This special issue reviewed AI developments and opportunities for each task of the cardiac imaging clinical workflow, surveying in detail cardiac imaging acquisition, segmentation and quantification, clinical decision support and precision cardiovascular medicine through integration with genomics data, in addition to ethical, social, and regulatory aspects. A close look at the statistics from Figures 2, 3, gathered from the publications reviewed in this special issue, shows a continuous increase in the research output in AI for cardiac imaging over the last 5 years. Interestingly, while cardiac image segmentation has received the most attention in the field as shown in Figure 3, due to the urgent need to accelerate the contouring process, it is also the only cardiac imaging task for which there is a decrease in the number of publications between 2018 and 2019 (Figure 2). This may be explained by the advances made possible by deep learning in the field, combined with an increasing interest from the community to invest in other AI applications such as cardiac image reconstruction or computer-aided diagnosis.

While this special issue described six main AI applications in cardiac imaging separately, we believe the ultimate aim should be to integrate these separate tasks into one single smooth, efficient and user-friendly pipeline for clinical cardiologists. As an example, this multi-task integration is the scope of the UK-based research project “SmartHeart: Next-generation cardiovascular healthcare via integrated image acquisition, reconstruction, analysis and learning” funded by UK's Engineering and Physical Sciences Research Council.

Additionally, while the special issue covered all important steps of cardiac imaging, it is worth mentioning other research tasks, at their very initial stages of development, which have not yet been covered in this special issue. For example, “automated quality control” is expected to enhance the cardiac image analysis workflow as large volumes of research and clinical data become available, and as the demand for AI-driven automation and robustness will increase in clinical practice. Here, it is worth listing a few preliminary works, such as automated quality control of CMR images using a deep learning approach to identify suboptimal image contrast or heart coverage (8). Other works have instead focused on quality control of the final image segmentation results using classical AI (9) or neural networks (10). Another area that may benefit from AI is “image-based computational cardiology,” which builds patient-specific digital models of the heart to simulate treatment response. While this area has been traditionally addressed using pure physiological and mechanistic models, researchers are now investigating the integration of machine learning to improve the accuracy and speed of the personalized simulated outputs (11). Furthermore, as larger datasets become available, it is expected that predictive models of disease progression will be developed and validated, including by integrating imaging with non-imaging predictors (e.g., socio-demographic, biomarker, lifestyle and genomic data). Finally, while this special issue is dominated by AI applications in echocardiology, CMR and cardiac CT, there have also been machine learning applications in “nuclear cardiology” (12), and these are expected to increase in the years to come as larger nuclear medicine datasets (both PET and SPECT) become available.

To conclude this editorial, we wish to emphasize the need in the next years for more concerted efforts dedicated to enhancing the technical and clinical advances described in this special issue, but also to address non-technical and non-clinical aspects of AI in cardiac imaging. Importantly, there is a need for community-defined standards and guidelines for validating and adopting future AI solutions, including metrics and procedures to evaluate performance, bias and errors, clinical effectiveness, degree of interpretability, and even cost-effectiveness. Benchmarking datasets and tools are also required to enable transparent and comparative analysis of the AI solutions across research institutions and players. For example, a test-retest reference dataset was recently compiled to assess reproducibility of machine learning CMR studies (13), while an international challenge on multi-center and multi-vendor cardiac imaging segmentation was organized to test generalizability across scanners (Siemens, Philips, General Electric and Canon)^1^. Finally, ethical and regulatory aspects will need to be established in a multi-stakeholder collaboration between experts in AI, bioethics and cardiac imaging, but also with the involvement of patient associations, private companies, and public authorities.

## Author Contributions

All co-authors discussed the structure and content of the special issue and paper. KL wrote the first draft. TL, AY, and SP revised the manuscript.

## Conflict of Interest

The authors declare that the research was conducted in the absence of any commercial or financial relationships that could be construed as a potential conflict of interest.

## Footnotes

^1^https://www.ub.edu/mnms/
